# Psychiatric comorbidity and psychosocial stressors among people initiating HIV care in Cameroon

**DOI:** 10.1371/journal.pone.0270042

**Published:** 2022-06-30

**Authors:** Angela M. Parcesepe, Lindsey M. Filiatreau, Peter Vanes Ebasone, Anastase Dzudie, Brian W. Pence, Milton Wainberg, Marcel Yotebieng, Kathryn Anastos, Eric Pefura-Yone, Denis Nsame, Rogers Ajeh, Denis Nash

**Affiliations:** 1 Department of Maternal and Child Health, Gillings School of Global Public Health, University of North Carolina at Chapel Hill, Chapel Hill, NC, United States of America; 2 Carolina Population Center, University of North Carolina at Chapel Hill, Chapel Hill, NC, United States of America; 3 Department of Psychiatry, School of Medicine, Washington University in St. Louis, St. Louis, MO, United States of America; 4 Clinical Research Education Networking and Consultancy, Yaounde, Cameroon; 5 Department of Epidemiology, Gillings School of Global Public Health, University of North Carolina at Chapel Hill, Chapel Hill, NC, United States of America; 6 Department of Psychiatry and New York State Psychiatric Institute, Columbia University, New York, NY, United States of America; 7 Department of Medicine, Albert Einstein College of Medicine, Bronx, NY, United States of America; 8 Departments of Medicine and Epidemiology & Population Health, Albert Einstein College of Medicine, Bronx, NY, United States of America; 9 Jamot Hospital, Yaounde, Cameroon; 10 Bamenda Regional Hospital, Bamenda, Cameroon; 11 Institute for Implementation Science in Population Health, City University of New York, New York, NY, United States of America; Kasturba Medical College Manipal, INDIA

## Abstract

**Background:**

Psychiatric comorbidity, the presence of two or more mental health disorders, has been associated with suboptimal HIV treatment outcomes. Little is known about the prevalence of psychiatric comorbidity among people with HIV (PWH) in sub-Saharan Africa.

**Methods:**

We conducted interviews with PWH initiating HIV care in Cameroon between June 2019 and March 2020. Depression, anxiety, post-traumatic stress disorder (PTSD), and harmful drinking were dichotomized to represent those with and without symptoms of each. Psychiatric comorbidity was defined as having symptoms of two or more disorders assessed. Moderate or severe household hunger, high anticipatory HIV-related stigma, low social support, and high number of potentially traumatic events were hypothesized as correlates of psychiatric comorbidity. Bivariable log binomial regression models were used to estimate unadjusted associations between psychosocial stressors and psychiatric comorbidity.

**Results:**

Among 424 participants interviewed, the prevalence of psychiatric comorbidity was 16%. Among those with symptoms of at least one mental health or substance use disorder (n = 161), the prevalence of psychiatric comorbidity was 42%. The prevalence of psychiatric comorbidity was 33%, 67%, 76%, and 81% among those with symptoms of harmful drinking, depression, anxiety, and PTSD, respectively. Among individuals with symptoms of a mental health or substance use disorder, a high number of potentially traumatic events (prevalence ratio (PR) 1.71 [95% CI 1.21, 2.42]) and high anticipatory HIV-related stigma (PR 1.45 [95% CI 1.01, 2.09]) were associated with greater prevalence of psychiatric comorbidity.

**Conclusion:**

Psychiatric comorbidity was common among this group of PWH in Cameroon. The effectiveness and implementation of transdiagnostic or multi-focus mental health treatment approaches in HIV care settings should be examined.

## Introduction

Mental health disorders are common among people with HIV (PWH) and are associated with worse HIV care continuum outcomes, including delayed engagement in HIV care, suboptimal ART adherence, and virologic failure [1–4]. Among the general population, psychiatric comorbidity, the presence of two or more co-occurring mental health disorders, is common and has been associated with greater psychiatric symptom severity and worse mental health treatment outcomes [5–7]. Similarly, compared to PWH with a single disorder, PWH with co-occurring mental health and substance use disorders have more persistent symptoms and worse mental health, substance use, and HIV treatment outcomes [8, 9]. However, research into the prevalence and correlates of psychiatric comorbidity among PWH in sub-Saharan Africa remains limited, and existing research has generally involved small samples [10–12]. One study in South Africa found that, among 17 PWH with at least one psychiatric disorder, the prevalence of psychiatric comorbidity was 29% (n = 5) [10]. A study of 200 pregnant women living with HIV in Tanzania found that 18% met screening criteria for depression and anxiety while a study of 44 PWH in the Gambia found that 32% met screening criteria for depression and post-traumatic stress disorder (PTSD) [11, 12].

Previous research has found psychosocial stressors, including material hardship, HIV-related stigma, traumatic life experiences, and low social support to be associated with poor mental health among PWH [13–19]. For example, results from a meta-analysis of studies conducted with PWH in Ethiopia found that the odds of depression among those with poor social support were 2.3 (95% confidence interval [CI]: 1.7, 2.9) times that among those with strong social support [15]. Similarly, among a sample of adolescents living with HIV in South Africa, the prevalence of anxiety symptomology among those with higher social support was 0.30 (95% CI: 0.13, 0.71) times the prevalence among those with lower social support [20]. A meta-analysis of the relationship between HIV-related stigma and mental health found that experiencing HIV-related stigma was associated with higher prevalence of depression [14]. Exposure to potentially traumatic life events has also been consistently associated with mental health disorders, including depression, anxiety, and PTSD [21, 22]. Despite established relationships between psychosocial stressors and poor mental health, little is known about the extent to which such psychosocial stressors are associated with psychiatric comorbidity among PWH in sub-Saharan Africa where psychosocial stressors are common and may present a substantial barrier to mental health and HIV care engagement and retention.

A better understanding of the prevalence and correlates of psychiatric comorbidity among PWH in resource-constrained settings can influence the development, implementation, and targeting of interventions to support the mental and physical health of PWH with psychiatric comorbidity and has the potential to improve HIV care continuum outcomes. For example, the Common Elements Treatment Approach (CETA), an evidence-based mental health treatment for depression, anxiety, substance use, and trauma- and stress-related disorders was developed with the purpose of treating individuals with co-occurring disorders [23]. In settings where psychiatric comorbidity is common, implementation of transdiagnostic or multi-focus interventions may be warranted.

The objectives of this analysis are to assess the prevalence of psychiatric comorbidity among PWH initiating HIV care in Cameroon and to characterize psychosocial correlates of psychiatric comorbidity. Specifically, this analysis investigates the extent to which four psychosocial stressors (household hunger, potentially traumatic events, anticipatory HIV-related stigma, and low social support) are associated with psychiatric comorbidity among PWH initiating HIV care in Cameroon.

## Methods

### Data collection

As previously described, data were collected from in-person interviews with 424 individuals initiating HIV care at three HIV treatment clinics in Cameroon between June 2019 and March 2020 [24]. Individuals were eligible to participate if they were 21 years or older and newly enrolling in HIV care at one of the three HIV clinics. Individuals transferring HIV care were ineligible for study participation. Data collection consisted of a structured interview conducted by a trained research assistant that included questions on mental health, substance use, psychosocial stressors, and sociodemographics. This study was approved by the Institutional Review Board at the University of North Carolina at Chapel Hill and the National Ethical Committee of Research for Human Health in Cameroon. All participants provided written informed consent.

### Measures

#### Depressive symptoms

Depressive symptoms were assessed with the Patient Health Questionnaire-9 (PHQ-9) [25]. The PHQ-9 is a 9-item screener that assesses the presence of depressive symptoms within the last two weeks. Scores for the PHQ-9 range from 0–27. Scores of 10 or greater are commonly considered an indication of a likely depressive disorder [25]. The PHQ-9 has been previously validated with PWH in sub-Saharan Africa [26–28].

#### Anxiety symptoms

Anxiety symptoms were assessed with the General Anxiety Disorder-7 (GAD-7) [29]. The GAD-7 is a 7-item screener that assesses the presence of anxiety symptoms within the past two weeks. Scores for the GAD-7 range from 0–21. Scores of 10 or greater are commonly considered an indication of moderate or severe anxiety symptoms [29]. The GAD-7 has been validated in a range of cultural settings, including among a primary care population with a high prevalence of HIV in sub-Saharan Africa [30–32].

#### Post-traumatic stress disorder symptoms

Post-traumatic stress disorder (PTSD) symptoms were assessed with the PTSD Checklist for DSM-5 (PCL-5) [33]. The PCL-5 is a 20-item screener that assesses the presence of PTSD symptoms in the past month. Scores range from 0–80. Scores of 31 or greater are indicative of probable PTSD. The PCL-5 has been validated across a range of cultural settings, including among a primary care population with a high prevalence of HIV in Zimbabwe [34–37].

#### Alcohol use

Alcohol use was measured using the 10-item Alcohol Use Disorders Identification Test (AUDIT) [38]. Scores range from 0 to 40. Scores equal to or greater than 16 were considered indicative of harmful drinking or potential alcohol use disorder [38]. This scale has been validated and used in populations with high HIV prevalence in sub-Saharan Africa [39, 40].

#### Psychiatric comorbidity

We created a dichotomous variable to represent individuals with and without symptoms of two or more of the following: depression, anxiety, PTSD, or harmful drinking.

#### Household hunger

Household hunger was assessed using the Household Hunger Scale [41]. This scale consisted of three questions about household hunger in the past 30 days: *was there ever no food to eat of any kind in your house because of lack of resources to get food; did you or any household member go to sleep at night hungry because there was not enough food; did you or any household member go a whole day and night without eating anything because there was not enough food*. If participants responded affirmatively to any question, they were asked about frequency of the occurrence. Responses indicating the event never occurred received a score of 0, responses indicating the event occurred “Rarely/Sometimes” received a score of 1, and responses indicating the event occurred “Often” received a score of 2, yielding an overall scale score ranging from 0–6. Scores of 2 and greater were considered indicative of moderate to severe household hunger [41]. This instrument was developed and validated for cross-cultural use to measure a household’s ability to access food within the last 30 days.

#### Potentially traumatic events

Lifetime exposure to twelve potentially traumatic events (PTE) was assessed, including experiences of physical and sexual violence, natural disaster, and the loss of a child, among others. One additional question asked individuals to identify any other PTE experienced during childhood or adulthood. The number of PTE reported was summed and categorized into quartiles based on distribution in the study sample (lower three quartiles = referent).

#### Social support

Social support was assessed using four items from the Multidimensional Scale of Perceived Social Support (MSPSS) that evaluated perceptions of social support from family and friends [42]. This scale has been previously validated with populations in sub-Saharan Africa [43, 44]. Participants were asked how much they agreed or disagreed with each of the following items: *I get the emotional help and support I need from my family; I can talk about my problems with my family; I can count on my friends when things go wrong; and I have friends with whom I can share my joys and sorrows*. Response options to each question ranged from strongly disagree to strongly agree. Responses were summed (such that higher scores were indicative of greater social support) and categorized into quartiles based on distribution in the study sample (upper three quartiles = referent).

#### Anticipatory HIV-related stigma

Anticipatory HIV-related stigma was assessed with 12 yes/no items created in accordance with the concept described by Earnshaw and Chadoir (e.g., if others know or suspect you are living with HIV, you might lose your job, your partner might leave you, your family members might treat you differently) [45]. A total anticipatory stigma score was constructed as the proportion of endorsed items from among all questions the participant was eligible to answer (i.e., participants without children or a partner were ineligible to answer related items). Anticipatory stigma scores were categorized into quartiles based on distribution in the study sample (lower three quartiles = referent).

#### Sociodemographics

Sociodemographic characteristics explored included age, gender, education, relationship status, employment, time away from home, and number of children.

Missing mental health symptom data. For individuals missing data on less than 10% of items for any given scale, the mean of the individual’s non-missing scale response was imputed for missing items.

#### Data analysis

Univariate analyses were conducted to assess the prevalence of psychiatric comorbidity in the study population overall. Bivariate analyses between each psychosocial stressor (household hunger, potentially traumatic events, anticipatory HIV-related stigma, and low social support) and psychiatric comorbidity were conducted using Pearson chi-squared tests among those reporting symptoms of at least one mental health disorder assessed. Separate bivariable log binomial regression models were used to estimate the prevalence ratios and 95% confidence intervals assessing the strength of the association of each psychosocial stressor with psychiatric comorbidity among those reporting symptoms of at least one mental health disorder assessed. Multivariable regression was not utilized as the aim of this analysis was to characterize marginal associations. All analyses were conducted with SAS Version 9.4 (Cary, NC).

## Results

Among the entire sample, over half of participants were female (58.5%), between 21 and 39 years of age (58.7%), and currently in a relationship (58.5%) (Table 1). Most participants were working for pay (64.4%) and had at least one child (81.3%).

**Table 1:** Sociodemographics and psychiatric comorbidity among 424 PWH initiating HIV care in Cameroon.

	Total N = 424	No MSD N = 263	One MSD N = 93 N (%)	2+ MSDs N = 68 N (%)	p-value
Age					0.36
21–39	249 (58.7)	149 (56.7)	55 (59.1)	45 (66.2)	
40+	175 (41.3)	114 (43.3)	38 (40.9)	23 (33.8)	
Gender					0.85
Male	176 (41.5)	107 (40.7)	41 (44.1)	28 (41.2)	
Female	248 (58.5)	156 (59.3)	52 (55.9)	40 (58.8)	
Relationship Status					0.11
Single	176 (41.5)	105 (39.9)	35 (37.6)	36 (52.9)	
Partnered	248 (58.5)	158 (60.1)	58 (62.4)	32 (47.1)	
Education					
None	30 (7.1)	14 (5.3)	8 (8.6)	8 (11.8)	0.20
Primary	218 (51.4)	131 (49.8)	52 (55.9)	35 (51.5)	
≥ Secondary	176 (41.5)	118 (44.9)	33 (35.5)	25 (36.8)	
Employment					
Not working for pay	151 (35.6)	95 (36.1)	28 (30.1)	28 (41.2)	0.34
Working for pay	273 (64.4)	168 (63.9)	65 (69.9)	40 (58.8)	
Away from home >1 month in past year					
No	261 (61.6)	170 (64.6)	55 (59.1)	36 (52.9)	0.18
Yes	163 (38.4)	93 (35.4)	38 (40.9)	32 (47.1)	
Number of children					0.96
0	79 (18.7)	49 (18.6)	18 (19.6)	12 (17.9)	
1+	343 (81.3)	214 (81.4)	74 (80.4)	55 (82.1)	
Missing	2	0	1	1	

MSD: symptoms of a mental health or substance use disorder

Among the entire sample, the prevalence of psychiatric comorbidity was 16% (Table 2). Among those with symptoms of at least one mental health or substance use disorder (n = 161), the prevalence of psychiatric comorbidity was 42%. The prevalence of psychiatric comorbidity was 33%, 67%, 76%, and 81% among those with symptoms of harmful drinking, depression, anxiety, and PTSD, respectively.

**Table 2:** Mental health and substance use disorder symptoms of 424 individuals initiating HIV care in Cameroon.

N (%)	
*Among the entire study population*	N = 424
No mental health outcome of interest	263 (62.0)
At least one mental health outcome of interest	161 (38.0)
1 disorder only	93 (21.9)
2+ disorders	68 (16.0)
*Among those with one or more mental health disorder*	N = 161
1 disorder only	93 (57.8)
2+ disorders	68 (42.2)
*Among those with depression*	N = 87
Depression only	29 (33.3)
Depression with 1+ comorbidity	58 (66.7)
*Among those with anxiety*	N = 54
Anxiety only	13 (24.1)
Anxiety with 1+ comorbidity	41 (75.9)
*Among those with PTSD*	N = 67
PTSD only	13 (19.4)
PTSD with 1+ comorbidity	54 (80.6)
*Among those with harmful drinking*	N = 57
Harmful drinking only	38 (66.7)
Harmful drinking with 1+ comorbidity	19 (33.3)

The prevalence of psychiatric comorbidity was higher among those who reported a higher number of potentially traumatic events (58.2% vs 34.0%; PR 1.71 [95% CI 1.21, 2.42]) (Table 3). Similarly, the prevalence of psychiatric comorbidity was 51.9% among those in the upper quartile of anticipatory HIV-related stigma compared to 35.9% among those in the lower three quartiles (PR 1.45 [95% CI 1.01, 2.09]). Although not statistically significant, the prevalence of psychiatric comorbidity was 53.5% among those in the lowest quartile of social support compared to 38.8% among those in the top three quartiles of social support (PR 1.38 [95% CI 0.96, 1.98]) and 50.9% among those with moderate or severe household hunger compared to 37.4% among those with no or little household hunger (PR 1.36 [95% CI 0.95, 1.95]).

**Table 3:** Psychosocial stressors and psychiatric comorbidity among PWH with symptoms of depression, anxiety, post-traumatic stress disorder, or harmful alcohol use who are initiating HIV care in Cameroon.

	Total N = 161	One MSD N = 93 N (%)	2+ MSDs N = 68 N (%)	PR (95% CI)^‡^
Household hunger^†^				
No/Little	107 (66.9)	67 (62.6)	40 (37.4)	1 (ref)
Moderate+	53 (33.1)	26 (49.1)	27 (50.9)	1.36 (0.95, 1.95)
Trauma				
Lower 75^th^	106 (65.8)	70 (66.0)	36 (34.0)	1 (ref)
Upper 25^th^	55 (34.2)	23 (41.8)	32 (58.2)	1.71 (1.21, 2.42)
Stigma^†^				
Lower 75^th^	106 (67.1)	68 (64.2)	38 (35.9)	1 (ref)
Upper 25^th^	52 (32.9)	25 (48.1)	27 (51.9)	1.45 (1.01, 2.09)
Social support^†^				
Lower 25^th^	43 (27.0)	20 (46.5)	23 (53.5)	1.38 (0.96, 1.98)
Upper 75^th^	116 (73.0)	71 (61.2)	45 (38.8)	1 (ref)

MSD: symptoms of a mental health or substance use disorder; PR: prevalence ratio; CI: confidence interval

^†^Missing: household hunger n = 1; stigma n = 3; social support n = 2

^‡^Modelling outcome of 2+MSDs (referent = one MSD)

## Discussion

The prevalence of psychiatric comorbidity was 16% among the entire sample population of PWH initiating HIV in Cameroon and 42% among those who screened positive for at least one mental health disorder assessed. Among those with symptoms of at least one mental health disorder assessed, the prevalence of psychiatric comorbidity was positively associated with anticipatory HIV-related stigma and experiences of trauma. Previous research with PWH in sub-Saharan Africa has estimated the prevalence of psychiatric comorbidity to be between 18–32% among PWH with at least one mental health disorder [10, 12, 46]. A study with PWH in South Africa found the prevalence of psychiatric comorbidity to be approximately 6% among the entire sample population (whether or not they had any mental health disorders assessed) [10]. It remains unclear why the prevalence of psychiatric comorbidity among this sample of PWH in Cameroon was higher than has been previously estimated. However, the current study assessed psychiatric comorbidity among PWH initiating HIV care. As such, we suspect that some portion of participants was newly diagnosed with HIV. The true portion of newly diagnosed participants in our sample is unknown, however, because date of first HIV diagnosis was not available for study participants. PWH newly diagnosed or initiating HIV care may be particularly vulnerable to psychiatric comorbidity, as HIV diagnosis has been found to be an acute stressor for many PWH [47]. Longitudinal analyses to examine the prevalence and persistence of psychiatric comorbidity following HIV diagnosis is needed. It is also worth noting that since 2016 Cameroon has experienced escalating political instability and violence as separatists seek independence for the country’s Anglophone regions. The mental health impact of this protracted conflict remains unknown. However, the mental health impact of protracted political instability and conflict has been previously established [48, 49].

Given that psychiatric comorbidity was common among PWH with symptoms of at least one mental health disorder assessed, the effectiveness of transdiagnostic or multi-focus treatment approaches for PWH and integration of such interventions into HIV care settings should be examined. Such approaches are designed to address common elements and mechanisms across mental health diagnoses and have been found to be effective when provided by trained, non-specialized providers in resource-constrained settings [50–52]. In addition, the effectiveness and durability of adaptive intervention approaches in which more and less intensive treatment approaches are targeted to PWH according to psychiatric need should be investigated. The extent to which evidence-based interventions developed to address one mental health disorder are effective in the presence of multiple mental health disorders also warrants investigation.

Psychiatric comorbidity was highest among those who screened positive for PTSD. Previous research with general populations has found that PTSD commonly co-occurs with other mental health disorders [53, 54]. Research into psychiatric comorbidity among PWH with PTSD remains limited. One study with PWH in the Gambia found that 32% met screening criteria for PTSD and depression [12]. Given that most participants with PTSD symptoms experienced psychiatric comorbidity, PWH with PTSD symptoms in Cameroon should be screened for other common mental health disorders. Transdiagnostic or integrated mental health treatment approaches that address common elements of multiple mental health diagnoses may be particularly appropriate for PWH with PTSD symptoms.

Our study found that psychiatric comorbidity was least common among those with harmful drinking. We are not aware of previous estimates of psychiatric comorbidity among PWH with harmful drinking in sub-Saharan Africa. Alcohol consumption is common among the general population in Cameroon, with 55% of men and 33% of women reporting recent alcohol use and 31% of men and 6% of women reporting recent heavy episodic drinking [55]. Cultural norms around alcohol use, including norms around drinking in recreational or social contexts, may influence our findings that harmful drinking commonly occurs in the absence of other mental health symptoms. Greater understanding of the context of harmful drinking among PWH in Cameroon could provide useful insight into why psychiatric comorbidity may be less common among this population.

Among PWH who screened positive for at least one mental health disorder assessed, having experienced a greater number of potentially traumatic events, compared to fewer, was associated with significantly greater prevalence of psychiatric comorbidity. Research into the relationship between psychiatric comorbidity and trauma among PWH is limited. However, our findings are consistent with research with a sample of Tanzanian adults with and without HIV that found that each additional traumatic event reported was associated with increased symptomology of both PTSD and depression [56]. Trauma has also been associated with suboptimal HIV care outcomes, including suboptimal ART adherence, increased viral load, and lack of viral suppression [21, 57, 58]. Research to understand to what extent mental health disorders or psychiatric comorbidity mediate the relationship between traumatic experiences and suboptimal HIV care outcomes is warranted. The extent to which trauma screening and the integration of trauma-focused interventions into HIV care improves mental health and HIV care outcomes should be investigated [59].

Among PWH who screened positive for at least one mental health disorder assessed, high anticipatory HIV-related stigma was associated with significantly greater prevalence of psychiatric comorbidity. This is consistent with research with pregnant women living with HIV in Tanzania which found that HIV-related shame was associated with screening positive for co-morbid depression and anxiety [11]. The mechanisms between anticipatory HIV-stigma and psychiatric comorbidity remain unclear. In addition, the relationship between other forms of HIV-related stigma, including internalized and enacted HIV-related stigma, and psychiatric comorbidity remains largely unexplored. Similarly, the relationship between psychiatric comorbidity and non-HIV-related stigma, including mental health-related stigma, stigma related to one’s sexual orientation or gender identity, or stigma related to engaging in sex work, could yield important insights. Research into the relationship between intersectional stigma and psychiatric comorbidity is needed.

Our findings support the need for strategies to address and prevent HIV-related stigma. However, evidence on the effectiveness of HIV-related stigma interventions remains mixed. Interventions that included both structural and individual-level components were found to be more effective than interventions with only individual-level components [60, 61]. While limited, there is evidence that mental health symptoms moderate the effectiveness of stigma-reduction interventions among PWH [62, 63]. An intervention with PWH newly entering HIV care in the U.S. found that depressive symptoms moderated intervention effectiveness, with greater decreases in internalized HIV-related stigma among those with higher levels of depressive symptoms [63]. Such findings suggest that stigma-reduction interventions may be particularly beneficial for PWH with mental health disorders.

Household hunger and low levels of social support were not associated with significantly greater prevalence of psychiatric comorbidity in regression analyses. We are not aware of previous research into the relationship between hunger or social support and psychiatric comorbidity among PWH. However, both hunger and low social support have been associated with poor mental health among PWH in sub-Saharan Africa [64–68].

This research should be considered in light of its limitations. All data were collected at entry into HIV care. As discussed, we were unable to distinguish between those who were and were not newly diagnosed with HIV. The relationship between psychosocial stressors and psychiatric comorbidity may differ at other points in the HIV care continuum and between those who were and were not recently diagnosed. In addition, data were collected from three urban hospital-based HIV treatment clinics in Cameroon and may not be generalizable to other populations or settings. Finally, the GAD-7, PCL-5, and AUDIT have not been validated in Cameroon.

## Conclusions

Psychiatric comorbidity was common among PWH entering HIV care in Cameroon with symptoms of at least one mental health disorder. Potentially traumatic events and anticipatory HIV-related stigma were associated with greater prevalence of psychiatric comorbidity. The effectiveness and implementation of transdiagnostic or multi-focus treatment approaches in HIV care to treat co-occurring mental health and substance use disorders among PWH should be examined in this setting. Future research should longitudinally investigate mechanisms through which potentially traumatic events and anticipatory HIV-related stigma are associated with psychiatric comorbidity and the extent to which these relationships persist throughout the HIV care continuum.
